# Supplementary material for: Factors associated with the timely completion of doctoral research studies in clinical pharmacy: A mixed-methods study
Source: PLoS One. 2022 Sep 30;17(9):e0274638. doi: 10.1371/journal.pone.0274638 (PMC9524630; doi:10.1371/journal.pone.0274638)
Supplement: S1 File — (DOCX) [file pone.0274638.s001.docx]

**Supporting Information**

**The following are the general interview questions**

1. Do you think that you finished your research at the expected time? Please explain.
2. At the beginning of the PhD programme, were you aware of the requirement of research publication before the award of the degree?
3. Did you have a timeline for your PhD research?
4. Was there any existing support system in the institution of the PhD programme?
5. Did you seek any form of support during the research process?
6. Did you seek any form of mentoring apart from your supervisors?
7. Is there anything you think should be improved upon in the PhD programme to facilitate the research progress and timely completion?
8. What is your advice to the newly enrolled PhD candidates?

**Interview 1**

R, I am going to interview you, I want to elicit some responses from you regarding your experiences as a PhD student programme.

Do you give your consent?

**R:** My consent is given already

**Question 1: Do you think you concluded this PhD programme within the expected time? Do you think you finished it within the duration you were supposed to finish it?**

**R:** No, because I am expected to finish it within three years but this is the fifth year.

**Interviewer:** because you did a full time study and not part time.

**R:** yes it wasn’t part time, it was a full time study.

**Interviewer:** what would have contributed to the delay in the completion of the programme

**R:** Actually, Covid contributed to it. At least one year was just lost due to COVID. So I can say it now 4 years, extra one year instead of 3 years.

**Interviewer:** So it was only COVID that caused it. Apart from the covid, you would have finished within the expected time?

**R:** Yeah apart from COVID, yeah, even minus COVID, I would have still been 4 years.

**Interviewer:** So apart from COVID, which other thing would have contributed to it?

**R:** The institution is also responsible for the extra one year.

**Interviewer**: Can you tell me how the institution is responsible

**R**: We had a delay, We started early 2017 but admission year was 2016. So it almost 3 years for us to be called for proposal presentation. There was no way we can start without presenting the proposal to the department. So we were joined with 2017. 2018. All of us were merged. SO we are highly disadvantaged.

**Interviewer:** Any other factor?

**R:** No other factor apart from this.

**Question 2: Did you work with a timeline? Were you able to work with a timeline?**

**R:** Yes I worked with a timeline because I was granted study leave to spend three years and come back. But noe the three years has elapsed. I have not even done the final defence. I finished my project waiting to be called for external defence which also took some months. This I was expecting that I will do by May but this is September.

**Question 3: At the beginning of the PhD programme, were you aware that it is important to have published a part of your work before you are awarded the degree, was it made specific to you?**

**R:** Yes we were told right from the orientation programme. We were aware right from the onset.

**Interviewer:** Did it put any extra tension on you? Were you like oh that is a challenge for me? How did you react to that?

**R:** Of course, I did not react, I am in academics already. Publishing has part of me. So it wasn’t really a problem to me.

**Interviewer:** so it wasn’t a big problem.

**R:** Yes

**Question 4: Which other challenges did you experience apart from what you have already mentioned that gave rise to the delay in the programme completion? were there other challenges you experienced?**

**R:** Challenges, I would say cost of the study. This is CKD. For CKD, everything is costly. Only laboratory investigation took up to a million Naira. So cost was a real challenge to me. And follow up. Because to follow up these patients is also very difficult because of the multi-functional involvement in CKD, very very difficult.

**Interviewer:** These challenges you mentioned now are specific to the research. Globally, as a whole, were there other challenges that you faced.

**R:** Yes even with my institution, I still had a challenge. I was supposed to be on full time study leave but I was not granted full time by the department. So I’m still carrying my full work and still running my PhD at the same time.

**Interviewer:** What was the reason?

**R:** The management gave study leave but the department said that we are short staffed. There was no way they can leave me. I think they should go by guideline, whereby one person will go, others should the person returns before another person will go. But because they can allow so many people to go at the same time while they allow others to go at the same time.

**Interviewer:** Were there other challenges that you had for eg. In the institution where you did the research?

**R:** In the institution where I did the work, I had no challenge because I had facilitators.

**Interviewer:** the few challenges you mentioned, how were you able to overcome them. Who did you meet, where did you go for support, was any support from the institution.

**R:** Yeah, from my own purse and the University where I work offered some assistance.

**Interviewer:** So you got a grant?

**R:** Yes.

**Question 5: Did you know of any existing support system in the institution where you did your PhD?**

**R:** Support system? My own institution or where I did the study?

**Interviewer:** Where you did the study.

**R:** Ok, for PhD students?

**Interviewer:** Yes.

**R:** Hmm, I’m not aware of that, nobody told us about that.

**Question 6: Did you seek any support from anyone apart from your supervisor for the PhD programme?**

**R:** Hmm,

**Interviewer:** Like experts in different areas

**R:** No, apart from my supervisor. Over there, we are still very young people,

**R:** Only my supervisor did it. Both as a supervisor and a mentor. Like two in one.

**Interviewer:** Very good.

**Question 7: Is there anything you think can be improved upon in PhD programme?**

**R**: What I will suggest is time management. So that we will be able to manage our time instead of having back logs. So that it will not be the fault of the students. Any student that want to graduate at a record time, should be able to do so. Not when you are ready, you will be begging the department to fix seminar.

**Interviewer:** so you experienced that problem. You were ready many times but the department was not ready.

**R:** Yes. Proposal level, I was ready but it took three years for us to be called for proposal presentation.

**Interviewer:** Is there any other thing you would like to say.

**R:** No there is no other thing.

**Question 8: What advice do you have for current PhD students?**

**R:** The advice is first and foremost, even before applying for a PhD, you should have an idea of what you want to do. That is the first thing. Because if a PhD student enrol in the programme not having an idea of what to do, that will cause unnecessary delay. That will not be easy between him and the supervisor. Just have an idea, then your supervisor can help you to filter the idea and now put it very well.

**Interviewer:** Any other advice?

**R:** No, I think this will suffice.

**Interviewer:** Ok. Thank you so much

**R:** Thank you too.

**Interview 2**

**Interviewer:** Do you give your consent for this interview?

**O:** Yes I do

**Interviewer:** Ok. Alright

**Question 1: So let me start. Do you think you finished your research at the expected time? The whole PhD programme, do you think that you are on time?**

**O:** Yes, I was on time. To the best of my knowledge, I was on time.

**Interviewer:** Ok. What do you think contributed to this timely completion of your programme? Can you tell me the factors or the things that you think helped or facilitated your completing on time?

**O:** Well the major factor that helped me was the rapport I had with my two supervisors, alright. They were more like family to me than teachers. So I could call them at any time of the day. Last year when I was collecting my data at the heat of COVID. I call them, sometimes I call them as late as 10pm, do you understand? And then because of that rapport and that freedom that they gave me, they also gave me the liberty to do my research. My two supervisors were there, they trusted me so much. I designed my work, I collected data, I did my analysis from the beginning to the end. So they gave me that trust. I believe that was part of what helped me to also complete on time. Because if I was waiting for someone to help me analyse, interpret and all these things, I think I would have spent more time than I did.

**Interviewer:** That means your knowledge of statistical analysis added to your completing your work on time, apart from the rapport.

**O**: Yes. Not only statistical analysis but designing a clinical trial. I had to undergo a course. Because of my work, I had to undergo a course by the University of Washington, USA on Epidemiology, alright. I had to take that course mainly to help me design this clinical trial because without that course, designing that clinical trial as a PhD student without experience in clinical trial, I would have made so many mistakes. Alright. If you check my work and if you check my proposal, you will find out that most of the mistakes I made in my proposal were averted. So that was what the course helped me in designing clinical trial.

**Interviewer:** Can you think of any other thing that contributed positively to your work?

**O:** Well another major factor that contributed was the Centre that I used for my work. I will say that it was conveniently sampled. In the centre that I used for my work, I had a high rapport with all the health professionals: the doctors, the nurses, the Med lab scientists, you know. They really helped me, the consultants, they trusted my judgements and my decision, they allowed me to make interventions. You know. And also, part of the trust was because I presented my Pharm D. so it gave them a little bit of confidence that I had experience in clinical practice. That I had an additional clinical degree. So I had high rapport with my study centre. They gave me liberty to handle patients. And then my patients were cooperative too. They also trusted my decision you know. If you tell them take this 2 g of salt, they whole heartedly take it trusting that it is going to be beneficial to their health. Then you are actually doing it for their own good. So I had the support of the centre I used and with the patients.

**Question 3 : Did you have a timeline for your PhD? Did you work with a timeline? Were there like mile stones you set for yourself?**

**O:** Of course. My study was an intervention. I was supposed to collect data every three three months.

**Interviewer:** I’m talking about the PhD as whole not just the research. You followed a timeline?

**O:** Yes

**Interviewer:** were you the one that marked it yourself or collaboration with your supervisor.

**O**: Definitely with my supervisor. My second supervisor Prof A…. when you finish your proposal, he will look at the proposal and ask you, how many years do you want to do this work and then you will tell him that you want to use two years to do this work. He will tell you that this is the work of 6 years. Before I landed at my final topic, he rejected, not really that he rejected but I presented 4 topics to him but when he will tell me that this study will take you like 8 years, I will just run away and go and bring another topic. But when he brought my topic, he said that this is achievable, it’s measurable and what you’re measuring are specific. So the name is SMART, something can be done within two years. That was part of the condition I had which encouraged me to continue with my topic.

**Question 2 : At the beginning of this PhD programme, were you aware of the requirement of research publication of research publication before the award of the degree, that you must publish something from your research before you’re awarded the degree?**

**O:** Yes. At the beginning of the programme, the PG school organized a course, PGC 701 for PhD students, we were told that we are supposed to present out work in international conferences, as an academic, that one is not a problem. They told us that starting from our set which was 2015, that we are expected to have at least one impact factor publication from our PhD work. So that was what encouraged me to write as I was collecting data, I was sending out articles for publication. I had it in my sub conscious that before my work will be sent out my work to the PG school that I would have an impact factor publication. As at 2019, as I was collecting data, I was already writing.

**Question 4: Was any existing support system in the institution you did your PhD programme?**

**O:** Any…?

**Interviewer:** any support for PhD students?

**O:** Like financial..

**Interviewer:** Any form of support system that will aid PhD students to start and finish their programme

**O:** There is no, I’m not aware of any support system in my own institution.

*Interviewer: But so far, things have been sounding so rosy, it looks too good to be true. I’m sure you must have experienced some challenges. Can you share some of your challenges?

***The above question emerged. It was not planned. So it can be included under the responses from those who did not conclude their programme within the expected time.**

**O:** I experienced a lot of challenges. In fact around May 2020, I just loaded credit on my phone and called one of my colleagues Dr. I… I was ranting for more than one hour, I told him that I was tired of this topic. Emotionally, the thing started affecting me, affecting my home, the time I spend for my children, my husband, affecting everything. It was as like everything was just falling apart. I will also say that it affected my social life. If you invite me for a programme, I will just calculate the time I’ll spend doing jamboree. And then if I invest that time on my lap top how much I was going to achieve. No no no, on a more serious note. It affected a lot of relationship. Some people were not understanding, you know. I didn’t have time to attend any event. I rather meet you and give you an envelope. It was draining financially. It affected me financially. I can say throughout my study programme sometimes I would be wondering what I was doing with my money, you spend without making budget because you want your work to be moving. It also affected so many things I wanted to do with money personally I couldn’t do them.

**Question 5 & 6 (merged): Did you seek any form of support, mentoring from someone else apart from your supervisors in the course of this your study? Especially in the institution where you worked or elsewhere?**

**O:** In the institution where I worked, I don’t know of any support. You support yourself

**Interviewer:** I meant in form of mentor, someone you go to apart from your supervisor, was any need for that throughout your work?

**O:** Not in my institution

**Interviewer:** Ok. But elsewhere

**O:** Yes. December 2019, somebody introduced me to international society for hypertension (ISH) and then I joined. You have to write an exam, you have to have a publication on hypertension to join. It’s mainly for researchers in hypertension. The person encouraged me to join because I have a lot to benefit. It is an international society for researchers in hypertension and then a subsidiary of the society is Women in hypertension, so I was also part of the society. In women in hypertension, once you just come in and say you have a need, before you even finish typing, somebody will stand up and ask you how can I come in? So the group was very supportive and then the ISH I had because of my age, if you’re less than 35, they will give free publications in the journal of hypertension. The journal of hypertension is Thompson Reuter’s journal. By God’s grace throughout last year, I had to utilize 4 publications before I reached 35 years. Their publication fee is 2,500 pounds.

**Interviewer:** So they waived all that for you, for your 4 publications?

**O:** Yeah, they waive it for it for you. They waive conference fee for you which is 2,000 pounds. So attend conference free.

**Interviewer:** Did you pay to join this group?

**O:** you write an exam, and go through an interview. But then during your interview, before your interview, you submit a publication or a research you already did in hypertension because they don’t want people that are not in hypertension. The society is strictly for research.

**Interviewer:** so that you don’t waste their resources.

**O:** yes. And then because I was dealing on factors affecting uncontrolled hypertension in Africa. Many people were interested in my work. People are so interested in uncontrolled hypertension. So many people interested in my work that it gave me … in fact one of the questionnaires that I used, that you have to pay big money to use, somebody helped me to get the syntax of that questionnaire for free. So I can say that the society really really supported me in my research.

**Interviewer**: Fantastic. Thank you so much. There a few more questions remaining. The other one has to do with ehh.

**Question 7: Is there anything that you think could be improved upon in the PhD programme in your institution to facilitate research progress and timely completion? Is there anything that is amiss that can be improved upon?**

**O:** It is difficult collecting study leave as a research student.

**Interviewer:** As a part time or full time?

**O:** as an academic and you’re running a PhD programme in the same institution. It is so difficult when you want to tell your HOD that you want to travel for 6 months to collect data. Anybody will roll eyes for you. If not for COVID-19 pandemic, I wouldn’t have finished my work by now. It would have been difficult for me collecting data. And most of the data, collecting data that you have to be there. You have people helping you. But if you are not there, by the time they give you the data they collected, you find out that the data is useless. They are not collecting the right thing. So that COVID-19 pandemic from Feb 2020 till November. Every day, I was going to the hospital even on Sundays. But if not for the COVID-19 pandemic, I don’t think my institution would have allowed me to travel from Feb to November. And without that everyday going to the hospital, I wouldn’t have finished collecting my data by now. So I think that even though we are part time students, our institutions can help grant study leave with pay. People are so afraid to apply for study leave because when you apply they say without pay. You need money to conduct research. So by granting students study leave, it will help students to complete their research.

**Question 8: Finally, the last question. What is your advice to current PhD students whether newly enrolled or those that are already in the system?**

**O:** my advice for newly enrolled students is just like I was advising two of my classmates at the faculty presentation this week B and Pharm A. last year when they stopped, I told them that you have to continue. Nobody is going to push you. You have to push yourself. Write down a goal. And then every day, you must be doing something. Every month you must be doing something. You may not be moving at that pace you want but then you are not stagnant, you are moving. So the most important thing is that you are moving. And once there is always a move one day you will finish your programme. Some of my class mates have not even gotten topic, some have not done proposal. You can imagine since 2015. One thing about PhD is that you can just get a topic, they say no, this is not a PhD topic. Or they will say this is not a clinical topic. Or it is an acute disease. So it can be so frustrating. So while you are waiting for your supervisor to get a topic for you, your supervisor has his or her own life. So I encourage every PhD student, once you have bought that form and have made up your mind to do PhD, keep on moving. Don’t stay one place for too long. If you’re frustrated or confused seek for solution. Yes look for solution look for senior colleagues or look for somebody that you can always harass. I always had people that I can go to and harass until they give you until they give you what you’re looking for. There is always need for that one person that you can comfortably run to, that will encourage you to keep on pushing. It’s something achievable. Once you set out to do it, it can be achieved.

**Interviewer:** Thank you so much Dr. O for your time.

**Interview 3**

**Interviewer:** I am about to start recording. Do you give your consent for recording?

**N:** Alright

**Question 1: “Do you think that you finished your PhD programme in due time? As in within the expected year or session? Let me put it that way.**

**N:** No.

Interviewer: Ok. What caused it? What do you think why you were not able to complete it within the time.

**N:** First of all, I didn’t have topic before I started. I didn’t have topic in mind. So we did course work. We were the first people that started course work. That course work took time. So after the course, I now started browsing and looking for topic, I wanted to deviate from my Masters work. So that took me time. Then strike added to the whole issue. The process of getting ethical clearance from various hospitals where I collected data is also there. Gwagwalada took more than a year to get their ethical approval. There was also strike in the hospital. At a stage, we started calling people that we know there so that I can collect my ethical clearance so that I can collect my data. Then collecting data is another baall game altogether. When you have to collect a lot of data, the people that have to get the data for you, some of them might promise to go to this, you find out that they didn’t go to get it, so you have to go there yourself. Then I was collecting my data from a different state where I was living. So I need to travel to the place and come back home. All that contributed to it.

**Question 2: At the beginning of the programme were you aware of the requirement of a publication from the research before the award of the degree? Was it explicitly made known to you that you must publish one article in an impact factor journal before the degree will be awarded to you?**

**N:** we were hearing it. I think that it also started in our set. It was not a requirement before, I think it was in our set that it was not written in the admission letter. That time we were hearing that even your Masters work has to be published. Later, we herd that it was no longer there, that it was only from your doctorate work.

**Interviewer:** did that put you under any tension whatsoever or was it something you braced up to and worked towards it.

**N:** it didn’t put me under any tension. In fact when I collected my first part of my work and had published it since 2019 in an impact factor journal. When I knew it, I worked to do that one.

**Interviewer:** apart from the things that you mentioned affected the completion of your programme within the due time. Were there other challenges as well that you would like to share?

**N:** All these things come with their emotional challenge. You have your own personal challenges, then the stress of doctorate is another stress. Waiting for this person, waiting for that person, all of them come with depression. Sometimes you get to the hospital to collect data, patients might not be in the mood. Like it was more difficult at National hospital at Gwagwalada, they said that a lot of people come there to collect data and the patients are like tired. It was more easier in the secondary hospital. What happened is that all these cause emotional stress to you. And then the finances. It is financially demanding. So if you don’t have a good source for funding the work, it will be a problem. I didn’t look into getting a sponsorship before I started and I didn’t have any guide. So nobody was mentoring me to do this or do this other one. So I was on my own per se. So having somebody to be there for you, telling you do this one or do that one can be helpful. So when I started working, the money that my work took from me is quite it took all the savings I had before I started the doctorate. I thought that by the time I have the doctorate I didn’t have any saving again. It crashed all the money that I had. So looking at the money you had saved over the years depleting like that comes with its own stress. But in all and all we give God the glory.

**Interviewer:** At least at the end you are seeing the results of your work.

**N:** I’m waiting for the PG school to approve my synopsis for the external.

**Question 3: But in all these, did you have a timeline, did you follow a timeline?**

**N**: Yes, I did, but I couldn’t finish within the timeline.

**Interviewer:** Ok.

**N:** So I stopped following my timeline.

**Question 4: Do you know of any existing support system in the institution where you did your programme? Was there any form of support in any way?**

**N:** Is it in UNN?

**Interviewer:** Yes in the institution where you did your PhD.

**N:** well I didn’t know of any

**Question 5: Where did you seek support when you had challenges? Who did you approach, I mean were there specific people you met apart from your supervisor that helped you through in this work, like mentoring?**

**N:** My colleague, my brother, even when I was reviewing my questionnaire I normally met some lecturers

**Interviewer:** In the department?

**N:** Yes. They helped me look at it. Sometimes, I talk to my brother who is a lecturer.

**Question 6: Do you consider these people as mentors? Do you think you had mentors apart from your supervisors during your work? Or there was nothing like that?**

**N:** I wouldn’t say that I had. Mentor is like a person you go with any problem you had and the person knows that you are his or her mentee. Right?

**Interviewer:** Yes, there is an agreement. It might be informal but somehow there is an agreement between both of you that there is that mentor-mentee relationship. Just like there is supervisor-supervisee relationship. Was there anything like that in the course of your research?

**N**: No, there was none. Just that if I have a problem I go to this person if not I look for a way to solve it myself.

Interviewer: Is there anything you think should be improved upon to facilitate research progress and timely completion?

**N:** I think that this whole process of synopsis they should find a way to rate down. If people should be presenting their work in stage like progress report. Not at the end of the work you start telling the person that your synopsis is not correct. Write this one, write the other one. You will be going up and down. If there can be people maybe 3 months of your work, 6 months of your work, people who sit on synopsis should be sitting on progress report so that they can be making any correction they have to make. Not when the faculty finished working another people will sit down and be giving another stress. If they think that the department and the faculty are not doing their work, they should start on time to do their own work. Because, I sent my synopsis for correction and has taken up to five months.

**Question 7: Is there any other thing you want improvement on?**

**N:** Yes. There should be research sponsorship for people. The university should support the students anyhow. Nothing is too small.

Interviewer: Do you have any idea of how that could be done. Because the University is saying that they don’t have any fund.

**N:** Faculties should have their research of interest according to their discipline. Like Pharmacy, pharmaceutical companies or hospitals. Or some people that will need the work that they are doing. So those people can sponsor.

**Question 8: Finally, what is your advice to people who are currently running their PhD?**

**N:** It’s a challenging time. Sometimes you are on your own. No one out there for you . So you must have an internal strength, your own internal shock absorber, because every day youre going out you don’t know what you will see. So you must have this inner strength within you. There is a time that you feel you must stop this whole and go and find another thing to do. Trust in God. Have faith.

**Interviewer:** Thank you so much N, I’m very very grateful.

**Interview 4**

**Interviewer:** I have started recording. I need to seek your consent for this interview. Do you give your consent?

**I:** Yes now.

**Question 1: Ok. Alright. Do you think you finished your PhD within the time you were expected to finish?**

**I:** No

**Interviewer:** Ok. What could have caused it. What led to the delay in the completion of the programme?

**I:** well the lock down number one, I will say distance too, after the departmental defence in Febraury, every other person around there was able to book their faculty presentation much earlier. Mine was fixed for June. But it coincided with my wedding. So I had to shift one. To a small extent my wedding.

**Interviewer:** which other thing can you think of?

**I:** And then getting a topic, being sure of your research is a very major reason, because coming in for the PhD, I didn’t what I was coming in to do. I wasn’t sure of a topic and then when I eventually got one, the feasibility study just showed me that I had to forfeit that. And changed the topic. So that took like a year. And then the nature of the research also.

Interviewer: What about it. How did it affect you?

**I:** Following up patients. Each patient you follow them up for 12 months. And getting patients with comorbid condition wasn’t so easy. I used 3 months to recruit them. And then came the follow up of each one for 12 months.

**Question 2: At the beginning of the PhD programme were you aware of the requirement of your research publication before the award of the degree? Was it made explicitly known to you that you must publish in an impact factor journal at least one article for your research work.**

**I:** Ok. Yes

**Interviewer:** Did that in any way give you any tension? Were you perplexed or was it a big challenge that you could achieve?

**I:** No I wasn’t in any way disturbed.

**Interviewer:** Because you were already in the academia.

**I:** Yes.

**Question 3: In all this, were you able to work with a timeline? Did you have a timeline at the beginning that you were trying to follow?**

**I:** The timeline for the research

**Interviewer:** A timeline for the whole programme including the research, it’s a whole package.

**I:** It extended beyond the anticipated duration**.**

**Interviewer:** Apart from the things you mentioned contributed to the delay of the PhD programme completion. Were there other challenges you had during your PhD research?

**I:** Before I got my approval from my school for a study leave. The course work was very stressful. So I was coming every week on Thursdays and Fridays. So Itravel from Uyo to Nsukka on Wednesday, then go back weekend to continue my work here before I was eventually granted study leave.

**Interviewer:** How long was the study leave, how many years did they give you?

**I:** Three years. But I renewed it after that to enable me finish the work.

**Interviewer:** Any other thing that you can remember that was a challenge for you?

**I:** I think I have mentioned everything.

**Interviewer:** Ok. If you remember any other one, you can always interrupt me while the interview is going on.

**I:** Ok.

**Question 4: Do you know if there was any support system where you did your PhD? Are you aware of any system to support PhD students in their programme?**

**I:** I don’t know if there is any.

**Interviewer:** But, in general how can you describe the support you got from the department. Was it what you expected, apart from the supervisor, the system, the structure

**I:** Yes, more than what I expected, very useful. Though I will always recommend in my department that school for PhD.

**Question 5: With these challenges that you had, did you seek any form of support from anywhere to be able to overcome them?**

**I**: well not really o. it wasn’t financial. Even if it was, who would you ask? Interacting with lecturers in the course of the programme, getting information, helped and talking to one or two peole in the hospital also helped me.

**Question 6: Somehow, you made use of mentors apart from your supervisors to enable you do this work. Can I put it that way?**

**I:** Well would I say mentors. I was just open to suggestions to whoever I spoke with because that is what I learnt when you are doing such programme, just listen to people, they lecturers, people in the hospital, clinicians, support staff in the hospital. They were all making suggestions.

**Question 7: In all this, are things that you would like to be improved upon (even though you would recommend it for people) things you would like to be put in place to facilitate the research progress of PhD students?**

**I:** The procedures

**Interviewer:** what about it?

**I:** The procedures, the departmental presentation, having to wait for some time, there should be a better system of presenting at the departmental level and knowing when next to present. And this synopsis issue. Let the people know how to write the synopsis well ahead of time and they say summary that we should know that it’s the synopsis you should follow. If there is a way of submitting to the department before the faculty presentation, better

**Interviewer:** So if there’s any error, you can start working on it at that time.

**I:** Yes

**Question 8: Finally what is your advice to current PhD students. What do you have to tell them?**

**I:** They should concentrate on their work. If they don’t have a research to look for one as soon as possible. Read wide, talk to people, observe, because you learn a lot by observing the things around you. Then whatever you have to present make sure you really understand it. Don’t rush, understand read wide, know what you are coming out to read. Then be open to suggestions, criticism, constructive ones I mean. With that you wont have any problem.

**Interviewer:** Thank you so much for your time and effort

**I:** you’re welcome.

**Interviewer:** Ok

**Interview 5**

**Interviewer:** Do you give consent for the interview?

**A:** Yes you have my consent. You can go ahead.

**Interviewer:** Also the interview is going to be recorded. Do you consent to that?

**A:** yes, I have consented to recording

**Question 1: First of all I will ask you the PhD programme you are about to finish, do you think you finished it within the expected time? Are you going to finish within the expected time?**

**A:** No. it is not because we registered for the 2016/2017 session and a three year programme so we are expected to finish by 2020.

**Interviewer:** Is it a full time programme?

**A:** Yes, it is full time

**Interviewer:** go ahead, what led to the delay?

**A:** The major reason for the delay is COVID-19 and ASUU strike.

**Interviewer:** Ok. That’s a major reason. Were there other factors that you think may have contributed to the delay?

**A:** I think these were the major reasons because without COVID-19 and ASUU I would have finished.

**Question 2: At the beginning of the programme was it made clear to you the requirement for the awarding of the PhD degree the publication from your research in an impact factor journal? Was it made clear to you?**

**A:** No it wasn’t

**Interviewer:** But later you got to know about it.

**A:** Yes

**Interviewer:** Did it put you under any tension, were you in any pressure when you got to know about that requirement?

**A:** Yes definitely, you know it was hard, I had to struggle to get the work submitted. Also most of these impact factor journals have financial implications.

**Interviewer:** So at the moment, do you have any work published?

**A:** No but at the moment two papers from my work are under review in two international journals that are impact that is the one accepted by the Univeristy.

**Question 3: Did you have any timeline for your PhD research? Did you work with a timeline or you were just doing at whatever happened let’s see. Did you have a plan?**

**A:** No because I was recently employed by my employer so definitely I had to finish within the timeline given to me by my employer. because of the delays I had to apply for an extension.

**Interviewer:** Apart from the factors that prevented you from finishing on time, were other challenges that you experienced with respect to the PhD programme that you want to share with us.

**A:** Well I think other challenges will be proper schedule for PhD especially from the University or the department. If there was proper, it helps students to finish on time.

**Question 5 & 6: Did you seek any form of support from anybody apart from your supervisor throughout this work? Were there people you could call mentors, people that helped you in one way or the other to be able to achieve a progress?**

**A:** Yes my PhD was more of collaborative work. I had help from the health care industry, you look at the consultants, experts in their own field especially the oncologist, honestly speaking, they had supported me with their expertise. The nurses also supported. Then if you come to the academicians, especially members of the panel, they also gave their own expertise. It is a bit collaborative because there are stakeholders.

**Question 7: I was going to ask you but you can add more things if you have. I was going to ask are there things you think should be improved upon with respect to the way the PhD programme is run in the institution where you did the programme?**

**A:** If proper scheduling is done, I think other challenges will be taken. Honestly speaking, from the department I did my PhD, I appreciate their support. But the schedule should be done at time intervals so that those who are ready can come and present to the house. And those who are not ready can meet up in the next one.

**Question 8: To end the interview, do you have any advice for current PhD students? People who are just enrolled or people who had been in the system?**

**A:** Honestly, my advice is that anybody who is coming to do PhD should first of all understand that PhD is different from any other degree programme. It is a programme that needs hardworking, patience and endurance. It will come with a lot of unforeseen challenges especially in clinical pharmacy where you are going to meet patients, going to meet other health care professionals, under normal circumstances you know how we are battling with them in the health sector, so you have pass a lot of obstacles before you are able to finish your work. Dedication, patience, knowing that all those are serious challenges especially making other health professionals that you know what you are doing and that you have something to offer then you will be accepted. Ah for the patients you need to professionally and ethically so that you will be accepted by the patients. For your work, you need to put a lot of time to read, read and read. Read very wide and consult your supervisors, and all other colleagues who can assist especially those who did clinical work.

**Interviewer:** Thank you so much Dr. A. I really appreciate your words of experience and all the time you have given to this interview.

**A:** All the best

**Interview 6**

**Interviewer:** Do you give your consent

**C:** Yes

**Question 1: I wont take much of your time. The first question is Do you think you are going to finish your programme in due time? Within the expected duration?**

**C:** Hmm I would say yes considering some factors.

**Interviewer:** Hmm Ok. Your programme is it full time or part time. How many years is the programme

**C:** Its part time. Its five years.

**Interviewer:** What are the factors that contributed to the timely completion of your programme? What do you think made it possible because

**C:** Majorly, what enabled me to complete within the five years was number one support, physical emotional and financial support. Both from my family members, my spouse, my supervisors and colleagues generally, people that could aid me in one thing or the other.

**Question 2: At the beginning of the PhD programme was it made clear to you the requirement of research publication before the award of the degree. Was it explained to you that you must publish before you get your PhD or you got to know about it later?**

**C:** No. we got to know about it later. But that guideline does not affect our set it is the set after. It was after our set that it became compulsory that we must publish from your PhD before completion.

**Question 3: Ok. Very good, did you work with a timeline for your PhD? Did you have like deadlines that you set for yourself?**

**C:** No I didn’t have a timeline, I couldn’t work with one

**Interviewer:** Did you experience any challenge during your PhD research or in general the whole programme.

**C:** Yes I experienced challenges. Some of them were in terms of family wise those were my own challenges, balance the home, balance and balance academic, those were my own challenges. But in the programme I think what delayed me a bit is preparing a research topic that one delayed me a bit but apart from that. In terms of course work, we finished it within one year

**Question 4: Do you know of any support that the institution gives to PhD students Is there any that you are aware of?**

**C:** In the faculty?

**Interviewer:** Yes where you did your PhD

**C:** There is no group support but you get support individually from people, some of them ask you how are you coping up with your PhD but in terms of group support there is none.

**Question 6: Apart from your supervisor were there people that you could call mentors that helped you in this programme?**

**C:** Yes

**Interviewer:** Who were they.

**C:** My supervisor before he became my supervisor and another colleague

**Question 7: Is there anything you think should be improved upon in the programme in the way the programme is run generally about the PhD in UNN or in pharmacy?**

**C:** I can talk in terms of Pharmacy I cannot say in terms of UNN but in terms of Pharmacy department, they should try to structure it to try to make it structures so that there is no delay

**Interviewer:** So that is what you want them to improve on, the structure.

**C:** They structure they have made they can improve on it and maintain it.

**Interviewer:** Ok. Is there any other thing.

**C:** I think another thing they can add is giving orientation within the department this will facilitate picking a research topic easily and faster. I think the challenges that PhD students have for their research work is to try and make their research in-depth because it can’t be the same as Masters work.

**Question 8: Then is there any advice you have for current PhD students or those who are planning to become one?**

**C:** I think the advice I have for those who are planning to start always get a research topic before you commence the programme I think it easier it makes it faster Once you are able to get a research topic before you come in to do the programme you can do the programme within 4 years but if you don’t have a research topic before coming in you tend to stay longer. Within that one year before the admission comes out if the person is smart enough should come to the department and meet anyone in the department and discuss with them you need to be able to come with research topic into to do it seamlessly.

**Interviewer:** Thank you very much. Is there any other advice?

**C:** There is always going to be challenges but try to balance the challenge of work life or family life with that of academic even though some have to take priority over the other. And try to be financially ready. If you are not financially ready then don’t bother because it will stagnate the work.

**Interviewer:** Thank you so much

**C:** You are welcome.

**Interview 7**

**Interviewer:** Before we start I need to get your consent. It is also been recorded. DO you give your consent?

**F:** Yes.

**Question 1: The first question I’m going to ask is with respect to the timing of your PhD programme. Do you think you finished within the expected time?**

**F:** No

**Interviewer:** Ok. What contributed to the delay?

**F:** Arriving at a topic, I didn’t arrive at the topic in time with my supervisor and then the duration of the research.

**Interviewer:** Ok it took more than you expected

**F:** Yes

**Interviewer:** Were some things that contributed specifically to the research taking longer time than expected?

**F:** The field experience is part of it you design your study but sometimes when you start it may not work out as you had planned. Sometimes in recruiting the patients.

**Interviewer:** Is it possible to itemize the challenges that you had in running this programme or put them in categories?

**F:** Choosing a topic, recruiting the study participants and writing up the report.

**Interviewer:** when you had these challenges, were the people you met to help you sort them out apart from your supervisor?

**F:** Yes other lecturers in the department where I am running the programme.

**Interviewer:** Other lecturers were equally important apart from your supervisor?

**F:** Yes

Interviewer: Then did you have a timeline for your PhD programme?

F: No

**Question 2: Also at the beginning of the PhD programme were you aware of the requirement of the research publication before the award of the degree? Was it made clear to you?**

**F:** No

**Interviewer:** It was something that came later on right? That you must publish before gaining the degree?

**F:**Yes

**Interviewer:** Did it put any tension on you? When you got to know about this requirement were you under any pressure

**F:** There was no pressure but it was an uphill task.

**Interviewer:** SO have you been able to publish anything from your work?

**F:** No

**Interviewer:** But is there anything that is under review, have you sent any for publication

**F:** Well I wrote an abstract somehow but due to one or two things it was not sent out.

**Interviewer:** SO do you have plans of sending them out before you conclude your programme.

**F:** Yes it is compulsory now except I don’t want to graduate.

**Interviewer:** What are the challenges you are having in that line or you are not having any challenges at all.

**F:** No there is no challenge

**Question 4: Ok. DO you know of any support system for PhD students in the institution where you are doing your PhD?**

**F:** I’m not aware of any.

**Question 7: Apart from the challenges you have mentioned with respect with the research were there also other challenges you had with respect with the programme structure the department? Were there things that were a bit disturbing that could have affected your work.**

**F:** Well I think the structure on ground of doing course work before research. If it is made open. That is as you come in, in fact making it in such a way that coming in for a phD work, you should have a topic already that you want to work, not you spend one whole year plus strike going to two years plus lectures after that’s when you start talking about topic.

**Interviewer:** Which other thing would you like to be improved on apart from what you have just mentioned now? Is there any other thing that you noticed durong the course of your programme you think is not right you something should be done about it in order to facilitate the future PhD students that will come in?

**F:** I think any other thing that I want ot recommend is if the system has a support system for PhD students.

**Interviewer**: What kind of support are you referring to?

**F:** Yeah, it can be material, it applies to finance. For instance you can have your good design but you don’t have a personal fund to run such a project. A good proportion of those who come for their PHd programme are not in academia. SO if such people have assistance either direct cash or maybe material it will also facilitate it. I think finance or material support in any way it can translated.

**Question 8: Finally, what advice do you have for people who are running their PhD currently or people who intend to come in?**

**F:** My advice is that they should solve the problem of choosing a research topic before picking up the form, a researchable gap you can fill and then as much as possible a close relationship between the supervisor and the supervisee because if a particular condition is not attached to graduation and bringing it up suddenly for instance for me when I heard it rather than get pressured I just slowed down, so its now publishing that is the issue because there are people who come for PhD are in the academia. So anybody whose promotion is not dependent on it simply means that the person had a flare for research in academic. Because that time your promotion is not affected. Unlike those in academia who that in two years you must be or another two years you must have published that kind of thing. For a system running PhD things should be made very clear for those coming in and then the mentee mentor relationship should be experienced as much as possible. Like me for instance a personal experience for my own department I enjoyed the relationship I had there.

**Interviewer:** Thank you very much Dr. F for your time and being able to say your experiences.

**Interview 8**

**Question 1: Do you think you finished the programme within the expected time?**

**E:** well no I didn’t finish within the expected time

**Interviewer:** what would have contributed to the delay in the completion of your programme?

**E:** Well the delay came from so many factors 1. From getting the ethical approval because I had to convince the medics who form almost 100% of the ethics committee that they did not have understanding of what pharmaceutical care is they felt that will mean interfering with their prescription and correcting their prescription without getting back to them. It took a while for me to convince them that Pharmaceutical care is a collaborating is like collaborating with the medics. Then the second delay was in recruiting the patients. I did a study on a disease that it highly stigmatized so for you to be able to convince the patient to come up and be part of the study. The ones that were enlightened had a lot of reservation because they felt that I work with a pharmaceutical company that I needed data from them. They had that reservation and you had to address that. Then there are so many things that you never expected in research. Research is something that is not straitjacketed. There are challenges that will come up that you have to address. Initially I designed my study to carry it out in 3 study centres but the logistics of collecting data from 3 centres was quite enormous so the supervisor guided me and said that I should reduce it to two. And I had to be there during clinic appointments so to my own favour, one of the clinic days was on Monday while the other was on Thursday. So I what I did was to be in one on Sunday a day before and then I travel to the other centre on Wednesday in order to collect data on Thursday. That a way protracted the study. But it’s ok. Plus other factors in school COVID came in and other bureaucratic issues in school.

**Interviewer:** Do you mind telling me in summary what are these bureaucratic issues in school?

**E:** Getting people to sign this that form and get them moved. Then to fix a date for one to present. And they said that only you cannot present. It is better to wait for the others so that it can be like a seminar. So all those hurdles. Had it been that as you finish you can present, it doesn’t work that way, you have to finish and wait for the others to be ready to present at the department and the faculty. COVID -19 and strike caused a lot of delay.

**Interviewer:** You have mentioned about 4 things now can you rate according to their order of importance in the way they impacted your research? Which one can you rate highest?

**E:** In terms of negative impact?

**Interviewer:** Yes

**E:** Ok well everything had its negative and positive sides. The negative side of the ethical approval delay made my protocol more robust because as they are raising those questions it made me better equipped for the job. There are things you anticipate already. Like when you see the response you will know these are the likely challenges that you will face when you get to the field. The questions they asked like the pharmaceutical care KAP I was able to easily answer them because they had come up during the ethical approval level. And again the delay in presentation helped me to go through my work over and over again and also got others to go through it for me to make it better. SO though there were challenges they also helped in a way.

**Interviewer:** So the COVID pandemic how did it affect your work?

**E:** The COVID pandemic delayed me. SO while waiting I was publishing. Also in writing the ethical review helps in writing your manuscript because when the reviewer makes corrections in your manuscript it also helps with the thesis. See you see, it also helps to reduce the number of questions that the external examiner will ask because those things have already been addressed. The COVID time helped me to think beyond the work. Not just to write and get your PhD but what happens after the PhD so it brought a lot of ideas. So what I should do post graduation. And really working on it after. So it helped.

**Question 4: Is there any existing support system in the institution where you did your PhD.**

**E:** Well on a personal level yes

**Interviewer:** Who are these people who gave you support apart from your supervisor?

**E:** Ok senior lecturers at the department, colleagues fellow students. I had a lot of support on a personal level yes there are.

**Interviewer:** So they helped you in terms of getting tools for your research

**E:** Yes in the designing of the work in the challenges I faced when I call they tell me that is how they did their own. Even in arranging the work before the presentation, I got a lot of support from lecturers.

**Question 6: Well do you consider these people as your mentors? What role can you give them because they are not your supervisor?**

**E:** well I call them .. the word mentor I see it as someone that you admire a lot and has helped you grow to be like him in a way. Somebody you see in this particular field of study, he picks you up and grooms you to be like him or even better than him. So I wont say mentors but teachers. Yes teachers because they taught me.

**Question 7: Is there anything you think should be improved upon in the PhD programme to facilitate research progress and timely completion for those who are running their PhD?**

**E:** Well yes in my experience, what I will say that clinical pharmacy most if not all about 80% of studies will be hospital-based. So the need for us in the academia to bridge that gap between the pharmacists in the academia and the pharmacists in the hospital because it is a huge gap. And also since we are talking about the patients, research with patients is team approach so we need to bridge that gap between the pharmacists in the academia and other health care workers because if we are able to bridge that gap a lot of collaboration will come in and make it a lot easier to do the PHD. Of course they will all be excited about the study and want to be part of it and it well you a lot. Then of course in this institution as well as in my own institution the emphasis is on the undergraduate. Everybody tries to ensure that the undergraduates finishes his or her programme on record time. But the post graduate programme is like you have to be the one to push. So if the post graduate student is not helped to graduate on time it has its own problem. If it is an MSc programme is time bound. For PhD if it is full time its 3 years and part time 5 years. So if someone comes for full time everybody should help the person to finish on record time. Not to leave the person to face all the challenges by himself. I had assistants a lot but it was on a personal basis but it wasn’t as if it was a structured.

**Interviewer:** Very good. Any other thing?

**E:** I appreciate the fact that courses have been introduced for PhD but what I have seen is like they are repeating what they taught in masters again. I someone is someone is based on pharmacoeconomics he should be taught pharmacoeconomics for 6 months or one year and he is grounded on that that is his PhD so it should lay emphasis on that. He had done research methodology already in Masters, Pharmaceutical care on Masters level, So if they are doing courses for him it should be tailored specifically towards his research area.

**Question 8: Thank you so much. Do you have advice for incoming PhD students.?**

**E:** Well what I would advice is that PhD programme is fully academic. You see people who come to do PhD not because they want to be academics but because they just want to do a PhD. For me I feel it’s a waste of time. If you don’t want to be an academic person , that you want to be in the academia, I don’t think you have any business with doing a PhD. You should love what you are doing. Because that will drive you even when you are facing challenges you tell yourself this is my area this is what I want to be I want to be an academic. But if you are in the hospital and say I want to do a PhD a essence is defeated. So if you want to do a PhD then you need ask yourself is that what I want to be and if the answer is yes, then why not.

**Interviewer:** Are you in the academia?

**E:** Yes I am.

**Interviewer:** Thank you so much Dr E.

**E:** Thank you too. I appreciate.

**Interview 9**

**Interviewer:** Dr Akunne I would like to interview you on your experiences regarding on your just concluded PhD programme. The challenge you had and how you were able to overcome them. The aim of the study is to evaluate the factors that contribute to research progress of PhD students. So the whole idea is to gain experience from those.

**Question 1: So in your estimation do you think you finished your research in due time? Your PhD programme was it concluded at the expected time?**

**M:** The expected time is five and I finished in 6 years so I will say I did not

**Interviewer:** So what contributed to not finishing on time?

**M:** Well I think I can vividly remember two. The first one was finance and the second one was based on the nature of the work like in my study, I did a systematic review and the search of the electronic data bases are partly the things that contributed to delay the work sort of. Because for me to get a collaborator that will have the research strategy was not easy. Because I searched electronic data bases currently in our library we are not able to do those searches effectively so I had to have a foreign collaborator so by the time I finished getting the contact to establish and all these thing I guess it took some time. The systematic review was the very first phase. And again the last phase which was also capital intensive and not just only that but getting trained data collectors too for the 5 tertiary facilities I used in the south eastern Nigeria. I needed to train them and get the questionnaires printed out. Some of these things were the challenges that contributed to the delay.

**Interviewer:** You mentioned finance, how were you able to overcome the financial challenge?

**M:** From my salary, I was saving and from other things I have

**Interviewer:** Was there any grant?

**M:** No there was no grant.

**Question 2: There is this issue of publication form the research as a prerequisite for the award of the PhD degree. Were you aware of it at the beginning of the PhD?**

**M**: I think the thing started with our set so we were aware. That timing will help because if you have divided your work once you finish one phase you can publish it so that at the end of five years you could have 2 or 3 publications.

**Question 4: Are you aware of any existing support system in the institution where you carried your PhD? Do you know of any for PhD programme?**

**M:** My husband mentioned something about TETFUND although I didn’t apply. Before I got to know it was very close to the deadline. I didn’t hear on time.

**Interviewer:** Is there any other support apart from finance?

**M:** Another thing was for staff in the form of subsidy. Maybe in the payment of fees. Which is not the same as non-staff.

**Question 5: Did you seek any form of mentoring or support from someone else apart from your supervisor in the institution where you carried your PhD?**

**M:** Hmm not necessarily in the institution where I did my study but from another institution like in the Nnamdi Azikiwe University Awka. I have a mentor there. Dr E… especially in the first phase he helped me. And in the institution where I carried out the study some of my colleagues were there. Do I need to say their names?

**Interviewer:** No, just mention the type of support they gave you with respect to the research.

**M:** Yes for the systematic review phase you cannot actually do it alone. You need at least 3 researchers especially in the data extraction phase. So I had colleagues that helped me in that. And like I mentioned before the search strategy was done in Germany by another collaborator and even in the course of the work I developed a questionnaire and another senior colleague also helped in data analysis too. So these are the people who helped apart from the supervisors.

**Interviewer:** So in your opinion there is need for someone apart from your supervisor to support PhD students like mentors.

**M:** Yes of course. Very much

**Question 7: Is there anything you think should be improved upon in the Phd programme in the institution where you carried out your PhD to facilitate research progress, timely completion anything?**

**M:** I think this issue of that grant should be well established. Although I think its captured but I don’t think it is effectively implemented in this University. Because I remember there is allowance for PhD students. If it can be given, it will help them in the finances a lot. Apart from subsidizing their school fees. Even once you register as a PhD student there should have a formal grant that will be for them. I believe it will help. Because for most persons finance is what is holding them especially those in the lab that need to buy so many chemicals and reagents. They can also give loan but grant is preferable.

**Interviewer:** Is anything you would like to be improved upon related to the PhD programme

**M:** To improve on the student-supervisor relationship helps because it will help the student to understand better and for the student to educate the supervisor because it is the student’s work.

**Question 8: What advice do you have for current PhD students?**

**M:** One important factor is that timeline is important. Make sure you are doing something. Because once you register, you have started. Try to see that you give time to everything, every activity of your work and work towards keeping that time. Time is very important. Make sure you do something every day. Because before you know it you finish it. But if you don’t feel like and keep it, it keeps piling up and after some time you will even forget where you are. Timing is a very important factor. If it is a five-year programme you divide it and know how you’re going to accomplish it and make sure that at every point in time you’re on time with the project you are going to carry out.

**Interviewer:** Thank you so much Dr, Akunne

**M:** You are welcome.

**Interview 10**

**Interviewer:** Like I had explained earlier the interview is about evaluating the factors that contribute to the research progress of PhD students. I am going to ask some questions that are bit personal. Feel free to express yourself.

**B:** It is ok.

**Question 1: In your opinion or estimation do you think you finished your PhD in due time?**

**B:** No

Interviewer; so what contributed to the delay in the completion of your programme?

**B:** Nature of the programme mostly

**Interviewer:** Ok what do you mean by that?

**B:** Nature of the programme and doing clinical pharmacy research in Nigeria that it population-based. And once it is population based you are dealing with human beings. And the complexity in human subjects is what contributed to all those problems. You start from dealing with the bureaucracy in the hospital. After that you get your collaborators and deal with their complexity. After that you start dealing with the complexity of patients. You recruit, you start getting data, you get drop out, you get a lot things. Also the expected outcomes that will measure the success or otherwise. It keep on changing. In my opinion, it is difficult for a PhD student in Clinical Pharmacy in Nigeria to start with five target outcomes and finish with those five targets. You will end up getting two out of the initial targets. So it’s the nature of the research. If it were any bench work or otherwise that I can determine my pace, get the materials, you will spend money, buy whatever I need, sleep in the lap take all the time, write publications, I am very sure I would have finished at least one year more earlier than I am finishing now. And also clinical pharmacy research in Nigeria is brain tasking or mental tasking. In the sense you pick a research area, you don’t see the methodology straight ahead of what someone has done before in Nigerian context. Take for instance, If I was in natural product whether chemistry or any where. I know that I just need to find a virgin or novel plant I need to follow the standard process of extraction and all these

Things, If I finish I need to isolate to do toxicity and I need to do that. So basically, you have that. But in my opinion, the challenges will force you to innovate and the weak man power within the country contributed to that. For now these are the challenges. And you know you cannot find a clinical study or trial in progress that you just key into take a part, get your outcomes, and go. Mostly you have to start your studies from the scratch which I know in some countries may not be like that. There may be clinical trial going on or a particular study that will last for years going on so you go you will be given a target your target is take this part follow these steps and finish up. Here you have to task your mind and your body, get the challenges stand up to the challenges get over them at the end of the day it may come out with some new things. But the good side of the challenges is that they make someone better so if that person is lucky to get through it well. The other bad side of it if care is taken. It can demoralize and for someone to get discouraged. That is my own opinion.

**Interviewer**: Thank you so much. Can you think of any other challenges you had with respect to your PhD programme?

**B:** Yes the challenges, I have not mentioned finance because whenever you are doing clinical studies involving patients, if you don’t have grants a huge amount of money to pay the patients to pay for the medications, pay for this pay for that definitely, you will have problems you have dropouts, it something that needs huge amount of money. But in Nigeria, we are treated the same with others. So you have to use your money wherever you cannot fund you have to be at the mercy of the respondents. So you have to pay people that will help you work, stay and monitor some parameters. Then when you get the data, in clinical pharmacy you have target to get only the clinical pharmacy skills. You have to get other skills if it is this area you are going into you have to dedicate yourself and learn it, a kind of unofficial residency. You learn a disease state, then you also learn some aspects of the analytics of the result. So all those things are part of the challenges. That makes the programme very bulky and tasking

**Question 2: At the beginning of the programme were you aware of the requirement of research publication before the award of the PhD degree? Was it made clear?**

**B:** During the post graduate seminar?

**Interviewer:** Yes

**B:** Yes I was aware

**Interviewer:** Have you been able to publish anything from your work?

**B:** So far I have 2 publication 3 conferences and more publications under peer review.

**Question 3: Did you have a timeline for your PhD did you work with a timeline?**

**B:** Yes, I did. I worked with a timeline. The first year was supposed to be my course work develop the research concept the second year I would have proposed and start going into the field. Even in the first year literature reviews were there, 2^nd^ year you would have review and search you would have developed the concept which I did then make a proposal which I did, then that 2^nd^ year to the middle of the 3^rd^ year the target was that I would have come out of the field. Got the data so the remaining half of the year is to package analyse and disseminating the results through seminars and publications. If you look at really for this whole programme is not that easy for a PhD student to do course in the first year, review literature and develop a good proposal all in one year. Most of them will go into the 2^nd^ year. Most of the people you see they end up making proposal end of the 2^nd^ year. So definitely by 3^rd^ year that is when you are finally in the field and then you start thinking that time is against you. So if care is not taken people will often approximate or shorten the time and that will affect the quality of the outcomes. So definitely you will see on the average if your research extend to that most people end of spending 4 academic sessions. Someone is lucky to graduate in 4 academic session may even end up paying the 5^th^ school fees because there are some aspects of maybe the external viva has entered the 5^th^ year. So if you look at it, the typical time given for a full time should have been 5 years so if it is 5 years and the standard of PhD is 3 years then there are problems. Number one ways of facilitating the programme has to come in for better and active supervision as soon as the PhD prospectus enter you have to guide him into starting work immediately even the student has to know not the someone will come blindly. The person don’t even know the area to go into after choosing area start looking for concept start reading. But I know with time with increase in clinical pharmacists in the academia, this will increase the supervision workforce possibly. But at the local level if I have a PhD student now I know its 3 years . we should rationalize those period like the first year these are the things to achieve. If we are doing things like , go and develop a concept, you are my student you are admitted, go and develop your concept with a timeline. What time between zero to how many months are you going to use for this By the 2^nd^ year how many publications are you expected. Are you publishing a review or whatever, so that the person will this is the timeline. So if I’m adding one year let me know the reason for the delay. The concept of designing the programme with respect to timeline and specific objectives mostly are not there. The other thing is that most PhD students are academic staff, they are employed. So 75% of your time is taken by your work and family responsibility and 25% for the PhD. That actually does not help, that is why those with the opportunity of going somewhere different from their own environment that is the best for them someone leaves and goes outside the country or a University that is not his own stay there full time that can also help.

**Interviewer:** so your programme was part time?

**B:** no it was full time

**Interviewer:** you were able to get a study leave?

**B:** Yes. I got study leave for the 3 years but we have to stay fully at work because we are the only ones teaching at the department. Even now that we came for the faculty and departmental seminar we just finished exams we were marking and compiling results so we just dropped it and came here. We arrived here and we continued with the preparation. So that is similar or the same thing with many academics in clinical pharmacy. Clinical pharmacy is an emerging area. You can’t afford not to work in the University. The University can give you study fellowship with the understanding that you would work. If you didn’t work who is going to work and train the undergraduates?

**Question 4: Was there any support system in the institution where you did your PhD?**

**B:** Honestly, the department, that is where we got all our support. We can’t quantify it. Personally from the supervisor and the department. We can tell them this is what we need and they will look at our job. They fix and design things for us and we are happy. Honestly, the department is welcoming. That is why it is not surprising that you keep on attracting more port graduate students.

**Interviewer**: so in your opinion there is always need for that support apart from the supervisors

**B:** Yes

**Question 5: And also did you seek support or mentorship from others apart from your supervisors?**

**B:** Yes, definitely. I worked on breast cancer I sought some support from the clinicians on the breast cancer therapy and cancer therapy generally. And I got it. I did residency in quote because I was around at the clinic and I learnt on the job. Also my work required high level of technique in data science. SO I had to learn and got some support primarily from my supervisor and secondarily from another person outside the department. So I had 2 sets of training and mentorship, Training of cancer therapy and training on data science.

**Question 7: Is there anything you think should be improved upon in the PhD programme to facilitate the research progress and timely completion?**

**B:** yes if you review our discussions those things we mentioned about the timeline for PhD especially in clinical pharmacy that 3 years is not realistic. So if people can finish it in approximately in 3 years it will improve the excitement in the programme. I know that within a short period of time I will improve and I will get the knowledge. So the department can innovate this issue. The PhD design of timeline. Between this time and this time you do this. Those who could meet with the timeline will automatically be dropped out. At least they know it’s their fault. Also the department can be following up we have at least 20 PhD students and what are they doing? Only five graduated. You keep reminding them what are they doing. Also the supervisors support. There is nothing that stagnates PhD more than the supervisors time. If the supervisor is so committed that he hardly looks at your work that will create a lot of problems. Like my work is ready for the past 6 months but my supervisor had not had time to look at it. Then confronting the challenges in the field you know when dealing with human beings and complexity. I know it’s not easy to say where to get scholarship but those study grants are very important because of their field work and they need money fund them officially or un officially these are important to do a very sound clinical research because lack of money.. when I designed my work, my work had three components or four; clinical outcomes, we have the pharmacoeconomics and the clinical pharmacokinetic aspect. I realized that I don’t have the money and time to go into the analytical technique and pharmacokinetsics. So I dropped the pharmacokinetics aspect because I wanted to use the plasma level outcome to see how it will influence clinical humanistic and economics indices. I couldn’t get that so I had to rely on the humanistic outcome and other things are made up/ you see for the quality of the work has reduced because I don’t have funding.

**Interviewer:** In your opinion do you think that the department can help the PhD students to get grants?

**B:** Yes the department can do that depending on the nature of the work. Honestly those grants are very important I’m seeing that the whole of clinical pharmacy, we have done a lot on patient education and safety and some intervention and establishing some relevance of clinical pharmacists. We have done a lot of that work pharmaceutical care intervention, impact of pharmacists led intervention. I think it is time for us to graduate and move to a higher level and those things will need grant and money. The reason why we will not go into the high level of clinical trial is because of the time the money and lack of grant. Any PhD prospectus has to take a problem that is realistic for him to solve. SO with that the department the faculty when we establish the some need for some of those trials I believe we can get grant and collaborate with companies that require studies like that. So if you get grant you can get 5 PHD students that will key into that grant they work and get their outcome. Like they do abroad. That should be the next phase. Because right now we have over flogged pharmaceutical care interventions in all the disease states, now the relevance of clinical pharmacists have been established. That I why ai was little bit rebellious to shift a little bit from the normal in my work

**Question 8: Thank you very much. One more thing before we go. What advice do you have for current PhD students those coming in and those already in it?**

**B:** Honestly, they used to say that PhD is not for everybody I now agree. If you don’t need it please don’t come. So that you will not have to suffer unnecessarily. That is one. Two, if you are not ready to task your brain and be innovative, please don’t come. If you cannot be hardworking, don’t come. Finally if you are not patient please don’t come. Those things you can’t take any of them away. You must be sure that you need the PhD.

**Interviewer:** Because sometimes because there is no job in the country let me get more degree. So who do you think needs PhD.

**B:** I came for PhD because I’m in the academics. I’m in the academics so I need the PhD . two I think that I can use the PhD outside the academic context. Three I know the passion I have. Those are the things that define my need. But for instance you have people that are director already in the hospitals that the PhD is not necessary for their promotion and they are not going into research but they just want to have the title so they will be battling with their work with all the challenges we mentioned they will be having a lot of seminars that are rejected and can be so demoralizing. As far as I am concerned the effort can be used in other aspects of the hospital practice that will help humanity. So make sure you need it. If you don’t need it. Don’t come. That is just my own honestly.

**Interviewer:** thank you so much

**B:** Thank you.
