# Supplementary material for: Factors associated with the timely completion of doctoral research studies in clinical pharmacy: A mixed-methods study
Source: PLoS One. 2022 Sep 30;17(9):e0274638. doi: 10.1371/journal.pone.0274638 (PMC9524630; doi:10.1371/journal.pone.0274638)
Supplement: Attachment — Submitted filename: Response to Reviewers.docx [file pone.0274638.s002.docx]

Hull York Medical School

University of Hull

Hull, United Kingdom

July 18, 2022.

The editor,

PLOS ONE.

Dear Sir,

Please find below my response to the academic editor and reviewers’ comments.

Best wishes,

Blessing Ukoha-Kalu

| Comment | Response |
| --- | --- |
| Please ensure that your manuscript meets PLOS ONE's style requirements. | Manuscript revised to meet PLOS ONE’s style requirements. |
| In your revised cover letter, please address the following prompts: If there are ethical or legal restrictions on sharing a de-identified data set | This has been addressed in the revised cover letter |
| Please relate the outcome of the study with author(s) country policy. | This has been added to the ‘discussion’ |
| The author only uses one public university as the sample. Please justify. | Phase one was conducted among current doctoral research students in accredited pharmacy schools in the south-east, south-south, south-west, north-central and north-west Nigeria. Phase two was conducted among doctoral students of the University of Nigeria, who recently completed their doctoral research program.  The public university used for phase two is one of the first universities that started postgraduate programs in clinical pharmacy. Also, the University records the highest number of doctoral students in Clinical pharmacy. |
| Please justify why the author(s) used 47 respondents. Is the sample meet based on your population. Please justify. | 47 respondents were used in phase one. In phase one, study sample = study population |
| The literature in the manuscript needs to be improved. Please add more literature about your independent and dependent variables. | This has been revised in the ‘introduction’ and ‘discussion’ sections. |
